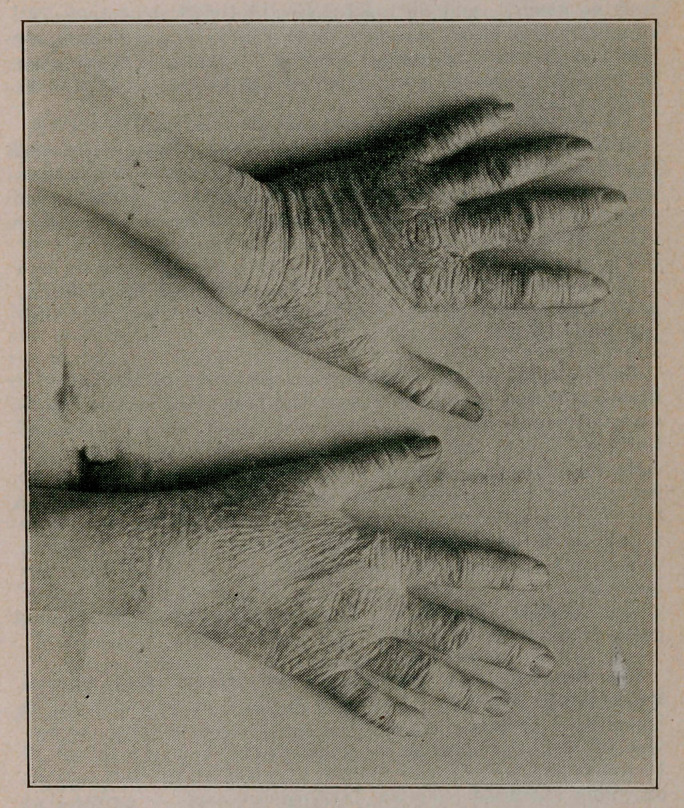# Pellagra

**Published:** 1914-07

**Authors:** 


### ABSTRACTS.

**Pellagra.** Dr. J. T. Windell, of Louisville, Louisville Monthly Journal of Medicine and Surgery, May, 1914, reports the case of a female, aged 33, a tobacco stemmer. The lesions healed under Fowler's solution, but returned. The condition had lasted 13 years. There was a gain of 25 pounds, disappearance of gastro-enteric symptoms, marked nervous symptoms had not occurred. Some doubt was expressed as to whether the case was not eczema. (The cut is reproduced through the courtesy of the author and the editor.)

## Figures and Tables

**Figure f1:**